# Diagnostic and treatment strategies in a memory clinic for older adults in a region with a predominantly low-income population

**DOI:** 10.3389/frdem.2026.1881788

**Published:** 2026-08-13

**Authors:** Patricia Solis, Ines Mintz, Nancy Medel, Zulma Sevillano, Julieta Lisso, Nicolas Irureta, Mauricio Federico Zalazar-Jaime, Brenda Giagante, Maria Carolina Dalmasso, Silvia Kochen

**Affiliations:** 1 Studies in Neuroscience and Complex Systems Unit (ENyS-CONICET-Universidad Nacional Arturo Jauretche-Hospital El Cruce), Florencio Varela, Argentina; 2 Memory Clinic, Atención Médica Integral (AMI), Hospital El Cruce, Florencio Varela, Buenos Aires, Argentina; 3 Department of Neuroscience, Hospital El Cruce, Buenos Aires, Argentina; 4 Facultad de Psicología, Universidad Nacional de Córdoba, Córdoba, Argentina

**Keywords:** Argentina, cognitive reserve, cognitive stimulation therapy, dementia, GeNED.ar, public health, vulnerable population

## Abstract

**Background:**

Early detection and interdisciplinary management of cognitive impairment are essential, particularly in socioeconomically vulnerable populations with limited access to specialized care. To address this need, a Memory Clinic was established in 2016 within the Argentine public healthcare system in Florencio Varela. This region reflects a condition of structural poverty, where 16.2% of the population lives in poverty according to Unsatisfied Basic Needs (UBN) and over 60% of residents lack essential services.

**Methods:**

The study included 1,204 individuals (852 women, 353 men) with a mean age of 72 years and 6 years of education, most of whom attended through self-motivation based on memory complaints. Based on cumulative experience, a work protocol tailored to the local population was established, comprising a cognitive battery (MMSE, ACE-R, FAB, CDR, FAQ, Zarit Scale, NPI-Q), neurological and functional evaluation, laboratory testing, and neuroimaging. Diagnoses are determined by consensus in weekly interdisciplinary meetings. Additionally, a cognitive reserve questionnaire was culturally adapted, implemented, and tested in a subpopulation of 315 people. Over the past three years, the clinic also contributed to the formation of GeNED.ar (Genetics and Neuroimaging in Aging and Dementia in Argentina), a national cohort study.

**Results:**

Evaluated patients received a definitive diagnosis along with guidance on healthy aging and, when necessary, appropriate pharmacological treatment or cognitive stimulation therapy. The implementation of the adapted questionnaire revealed a significantly lower cognitive reserve in patients with dementia. Furthermore, active participation in research projects like GeNED.ar demonstrated enhancements in clinical care, supported staff training, and promoted alignment with international standards.

**Conclusion:**

As the only public healthcare center of its kind in the region, this Memory Clinic provides high-quality, research-integrated cognitive care to a vulnerable elderly population. This center offers a successful and replicable model for similar socioeconomic contexts across Latin America.

## 1 Introduction

Dementia is a clinical syndrome characterized by progressive cognitive decline that interferes with daily functioning and significantly impacts quality of life. According to the World Health Organization, more than 55 million people worldwide live with dementia, to rise substantially in the coming decades due to population aging (World Health Organization, 2021). Alzheimer’s disease accounts for approximately 60–70% of dementia cases, followed by vascular dementia, frontotemporal dementia, and dementia with Lewy bodies (Alzheimer’s Disease, 2022). This etiological heterogeneity complicates diagnostic pathways, which place increasing pressure on healthcare systems (Dickerson et al., 2025), particularly in low- and middle-income countries (LMICs), like Argentina.

Despite broad consensus on the importance of early detection and multidisciplinary management (Bagnati et al., 2025; Dodich et al., 2025), access to timely diagnosis and appropriate care remains highly unequal. Structural barriers such as limited availability of specialized services, low educational attainment, cultural stigma, and fragmented healthcare systems frequently delay diagnosis, especially in socioeconomically vulnerable populations (Livingston et al., 2020a, 2020b; Parra et al., 2021). These challenges are particularly evident in the Memory Clinic running in the Hospital El Cruce (HEC) outpatient clinic Asistencia Medica Integral (AMI), which provides care exclusively to elderly people with Programa de Atención Médica Integral (PAMI) coverage. PAMI is the largest state-run program for disabled and senior citizens, broadly comparable to Medicare in the United States (Palacios et al., 2020). The Memory Clinic is located in Florencio Varela, where a government report showed that 20% of the population lives in census areas ranking lowest on the Multiple Deprivation Index, with an average of 0.36, ranging from 0 to 1.^1^ Some observations made a few years after the Memory Clinic was created, were that 29.5% of attendees were functional illiterates (<3 years of schooling) and 39.7% only completed primary school (Kochen et al., 2018). This low level of education increases the complexity of neurocognitive assessment and diagnosis. In addition, there was a high rate of misdiagnoses, with 25% of the population without dementia being medicated with drugs usually used for dementia, and more than half of people living with dementia were not treated (Kochen et al., 2018). Surprisingly, people with very low cognitive scores had a certain degree of autonomy, performed some of the daily activities and were involved in community life, proving the importance of functionality concept (Kochen et al., 2018). These particularities of the population highlight the necessity to develop tools that allow these people to receive a formal diagnosis and appropriate follow-up.

In this work we describe the clinical and methodological evolution of the Memory Clinic within the public healthcare system, and we report population’s characteristics and outcomes. Evidencing our commitment to rigorous scientific research and the production of knowledge that translates into improved clinical practice.

## 2 Methods

### 2.1 Study design and population

A retrospective study was conducted between January 2016 and January 2026. Study participants comprised individuals attending the Memory Clinic (HEC-PAMI) located in Florencio Varela, Argentina. Outpatients aged 60 years and older, without cognitive problems, with mild cognitive impairment (MCI), with Alzheimer’s-type dementia or any related dementia were included. Individuals with other neurological or psychiatric diseases were excluded from the analysis. The study was approved by the Research Ethics Committee of the HEC. The access to retrospective data used for demographics was approved by the Research Ethics Committee of the HEC. On the other hand, the GeNED.ar study protocol was approved in 2023 by the same Research Ethics Committee (D0072). Written informed consent was obtained from all participants or their family members/legal tutor if cognitive impairment precluded consent.

### 2.2 Standardized clinical assessment and management protocol

This standardized protocol was set and applied to all participants in this study and is currently applied to all patients evaluated at the Memory Clinic. At the initial visit, a directed anamnesis was conducted with both the patient and cohabiting relatives or caregivers, covering cognitive symptoms, behavioral changes, functional performance, and social context. This was followed by a comprehensive neurological examination and functional assessment. Laboratory tests, neuroimaging studies, and standardized neurocognitive batteries were subsequently requested to complete the diagnostic workup. Diagnoses were established through consensus among Memory Clinic professionals, integrating clinical, cognitive, functional, laboratory, and neuroimaging findings in accordance with the criteria proposed by McKhann et al. (2011). All patients received evidence-based lifestyle counseling aimed at supporting the prevention of cognitive decline and dementia. When clinically indicated, pharmacological treatment (Schmidt et al., 2015; Profyri et al., 2022) and or referral to a culturally adapted cognitive stimulation therapy was prescribed, and patients were enrolled in a structured program of periodic neurological follow-up.

### 2.3 Cognitive and functional assessment

**Figure 1:**
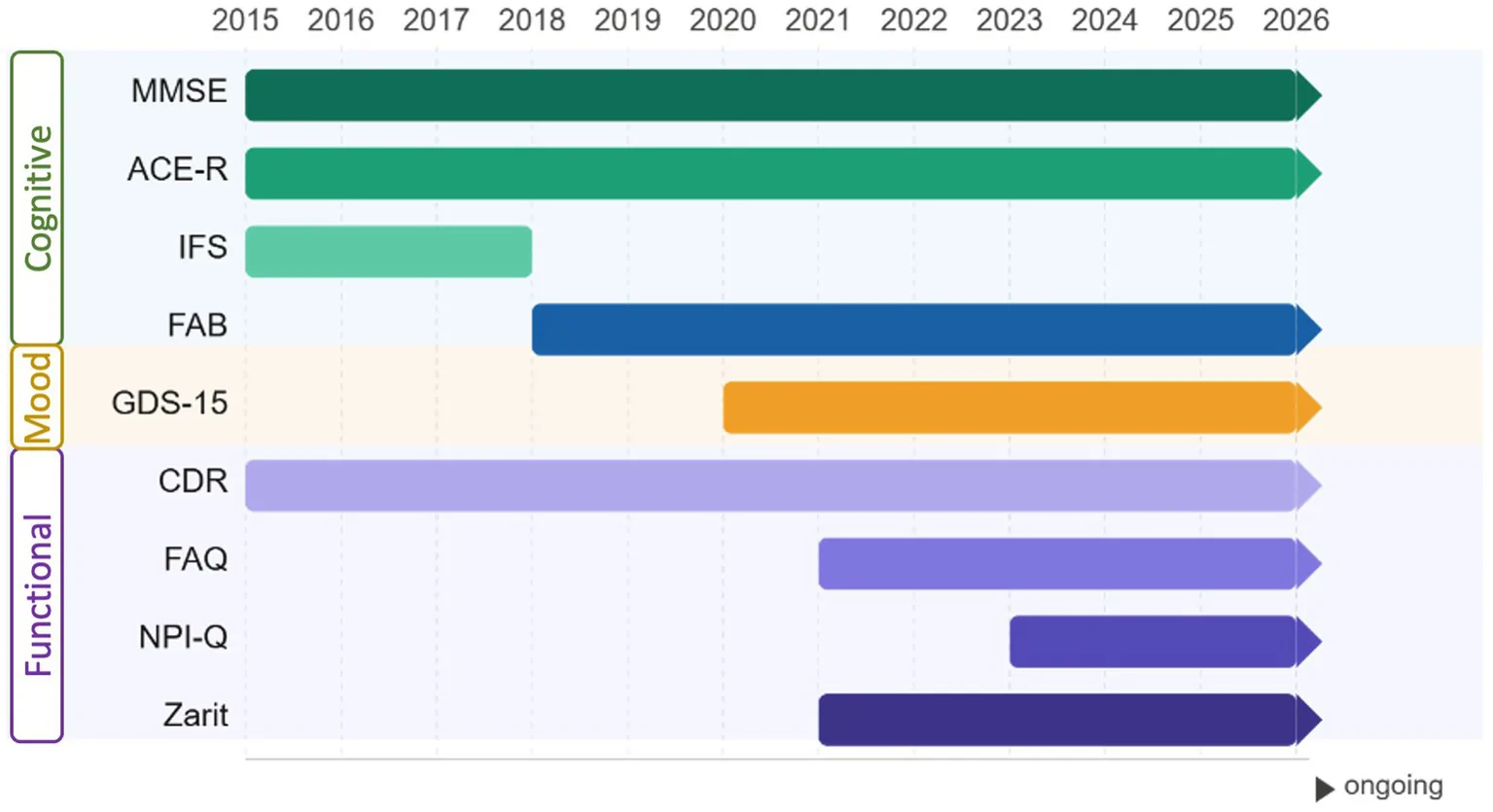
Neuropsychological assessment protocol timeline. The timeline illustrates when each cognitive test was included in the evaluation protocol from the beginning of 2016 to the end of 2025. Within the blue background are cognitive tests: MMSE (Mini-Mental State Examination, Rio de la Plata version), ACE-R (Addenbrooke’s Cognitive Examination-Revised Spanish version), IFS (INECO Frontal Screening), and FAB (Frontal Assessment Battery Spanish version). The yellow background includes depression estimation by GDS-15 (Geriatric Depression Scale). The purple background includes tests to estimate functionality and dependence: CDR (Clinical Dementia Rating), FAQ (Functional Activities Questionnaire), Neuropsychiatric Inventory Brief Questionnaire (NPI-Q-Spanish version), Zarit (Zarit Burden Interview).

The cognitive evaluation protocol comprised the Mini-Mental State Examination (MMSE)-Río de la Plata version, using age- and education-adjusted cut-off scores as described by Butman et al. (2001), and the Addenbrooke’s Cognitive Examination-Revised (ACE-R) Spanish version (Torralva et al., 2011), with specific cut-offs according to years of educational attainment (Sousa and Vivas, 2017). Executive function was assessed by the INECO Frontal Screening (IFS) (Zapata-Zabala et al., 2019) and the Frontal Assessment Battery (FAB)-Spanish version (Hurtado-Pomares et al., 2018). Furthermore, the Geriatric Depression Scale (GDS-15) (Martínez de la Iglesia et al., 2002) was administered to screen for depressive symptomatology based on clinical observation. To assess functionality, dependence, and autonomy, caregivers completed the Clinical Dementia Rating (CDR) (Hughes et al., 1982), the Functional Activities Questionnaire (FAQ) (Pfeffer et al., 1982), the Neuropsychiatric Inventory Brief Questionnaire (NPI-Q)-Spanish version (Boada et al., 2002) to identify significant behavioral changes, and Zarit Burden Interview (Zarit et al., 1980) to evaluate caregiver strain and provide necessary support. These tools were included in the protocol at different timeline points since 2016, shown in Figure 1.

### 2.4 Cognitive reserve

To assess cognitive reserve, we used the Cognitive Reserve Questionnaire (CRQ) (Rami et al., 2011). This instrument evaluates three key dimensions: educational background (years of schooling, additional courses completed, and parental education level), life history (occupational history and language proficiency), and lifestyle habits (frequency of reading, engagement in mentally stimulating activities, social participation, and physical activity). Its brief administration time and accessible language make it particularly suitable for populations with low levels of formal education and for implementation in public health settings with limited consultation time. Cultural adaptation of the cognitive reserve questionnaire was performed according to the methodological recommendations available in the literature for cross-cultural adaptation of assessment instruments. The detailed description of the adaptation process and the psychometric properties of the adapted version will be presented in a separate manuscript, which is currently in preparation. Nevertheless, preliminary findings suggest that the instrument exhibits adequate psychometric characteristics for its application in the target population.

### 2.5 Culturally adapted cognitive stimulation therapy (CST)

Cognitive Stimulation Therapy (CST) is a structured group intervention originally developed in the United Kingdom, consisting of activities and discussions designed to improve cognitive and social functioning in people with dementia (Spector et al., 2006; Orrell and Spector, 2017) CST was adapted to the local population and implemented at the Memory Clinic as described in Medel et al. (2024). Briefly, the CST manual (Spector et al., 2006), was translated to Spanish and sent to the international CST team for revision. Cultural adaptation followed all phases of the phases established by the Formative Method for Adapting Psychotherapies (Aguirre et al., 2014), involving structured interviews with an interdisciplinary group of participants and informants, including health professionals from cardiology, traumatology, diabetology, pulmonology, and nursing; twelve family caregivers; ten patients with Alzheimer-type dementia; and the Memory Clinic neurology and neuropsychology team. For implementation, a successful CST pilot study was conducted including patients attending the Memory Clinic with a diagnosis of mild dementia and/or MCI (Medel et al., 2024). Currently, is carried out twice per year.

### 2.6 Neuroimaging

For diagnostic purposes, computerized tomography or magnetic resonance scans, at any resolution, were accepted and integrated into the diagnostic protocol. However, patients were encouraged to undergo scanning at the HEC facility, where a 3 Tesla Philips Achieva scanner is available, allowing the Memory Clinic team direct access to imaging results. The standardized imaging protocol included high-resolution structural imaging (weighted T1), diffusion-weighted imaging, resting-state functional MRI, and weighted T2 and Fluid Attenuated Inversion Recovery (FLAIR) sequences.

### 2.7 Statistical analyses

All statistical analyses in this manuscript were performed using R-project (R Core Team, 2025). T-test was used for two continuous variables comparison like age and schooling, chi-square test was used for discrete variables like sex and diagnosis, logistic regression models were used to evaluate impact of sex in cognitive impairment at first visit. Data plots were obtained with the package ggplot2 (Wickham, 2016).

## 3 Results

### 3.1 Consolidation of the memory clinic and establishment of a standardized care protocol

The Memory Clinic was founded in 2016 in response to a critical gap in access to specialized neuroscience care for PAMI-enrolled older adults residing in Florencio Varela, a southeastern district of Greater Buenos Aires, Argentina; a population characterized by high levels of housing deprivation, and labor informality [Sistema de Información, Evaluación y Monitoreo de Programas Sociales (SIEMPRO), 2019]. Prior to its implementation, beneficiaries in this region faced significant barriers to accessing specialized neuropsychological evaluation, limiting primary care physicians’ capacity to establish accurate diagnoses due to the absence of specialist support (Kochen et al., 2018). Building on the findings reported in that initial publication, the neuropsychological assessment protocol was subsequently refined and expanded (Figure 1) to match population needs. Over the study period, the clinic consolidated from addressing a previously unmet regional need into an established referral center for cognitive disorders, currently attending approximately 2,160 ambulatory patients per year. The clinic’s integration into the public healthcare network represents not only an expansion of specialist access but also a strategic response to the compounded health risks associated with social vulnerability, as defined by World Health Organization criteria (World Health Organization, 2019). Access to neurological care within the PAMI system requires a primary care referral. Although all patients met this requirement, the decision to seek care appeared to be predominantly driven by patients or their relatives, rather than by a formal clinical indication. In response to this clinical reality, a standardized assessment and management protocol was developed and implemented, as described in the Materials and Methods section.

### 3.2 Cognitive assessments battery optimization

The current neuropsychological battery was established based on the characteristics of the population. MMSE and ACE have different cut-offs based on years of schooling. In addition, at the beginning of 2019, the IFS was changed to the FAB to evaluate frontal activities (Figure 1). This transition was strategically implemented to better suit the cohort’s sociodemographic profile, since FAB tasks are less complex and it is easier to administrate than IFS, and it is validated and used world wide. Functionality was initially assessed only by CDR scores. In 2021, FAQ was incorporated into the protocol to complement the CDR and provide a more comprehensive functional profile; which was additionally enriched in 2023, by incorporating the NPI-Q adapted Spanish versions (Figure 1) to identify significant behavioral changes. Additionally, the Zarit interview was also introduced in 2021 to evaluate caregiver strain and provide necessary support, and the GDS-15 was added to the protocol in 2022, to screen for depressive symptomatology based on clinical observation and patient interviews, to enhance diagnostic depth. Notably, data collection from informants was occasionally limited, as several patients attended appointments unaccompanied or caregivers faced difficulties completing the instruments (Figure 1).

### 3.3 Characteristics of the population attending the memory clinic

**Figure 2:**
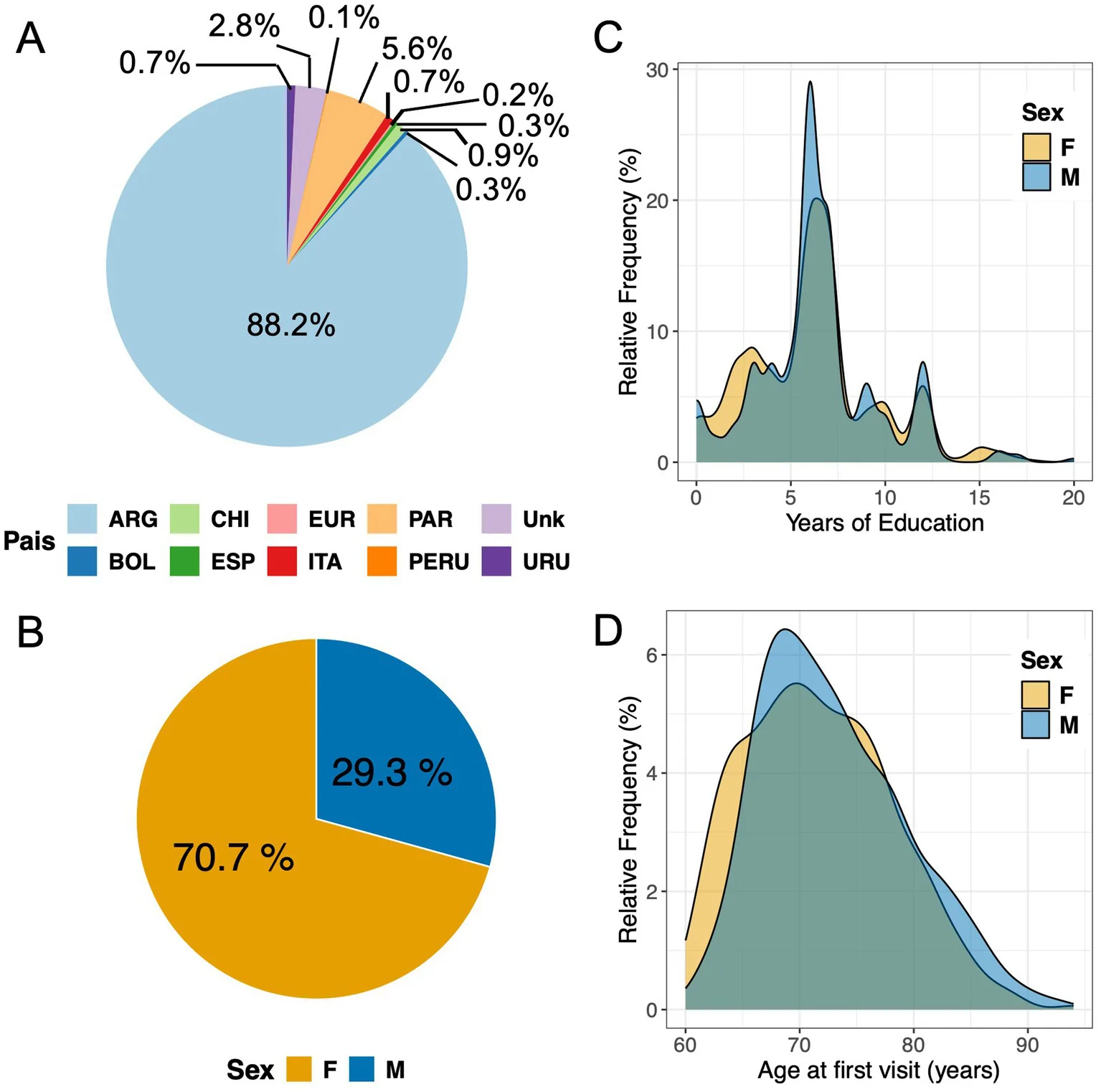
Population characteristics: nationality of people attending the Memory Clinic **(A)**; biological sex proportions in the sample **(B)**; density plot of education in years, stratified by sex **(C)**; and density plot of age distribution, stratified by sex **(D)**. In **(A–C)**, sex is female (F) and male (M).

Over the past ten years, data were collected from 1,204 patients attending the Memory Clinic, all residing in Florencio Varela and surrounding areas. Notably, 11.8% were immigrants, reflecting the demographic complexity and diversity of this population. The majority of immigrants (8.6%) originated from neighboring countries, with Paraguayans representing the largest subgroup (5.6%), while 1.5% were of European origin, predominantly Italian (0.75%); country of origin data were unavailable for the remaining 2.8% (Figure 2A).

Consistent with our previous observations (Kochen et al., 2018), women comprised 70.7% of the sample (852 women and 353 men, Figure 2B). The overall educational level was low (Figure 2C): 22.2% of patients had three or fewer years of formal schooling, and only 3.3% had reached university level, with no statistically significant differences between sexes (*p* = 0.43). Most patients lived with family members, and fewer than 5% presented indicators of social isolation.

The mean age of the sample was 72.1 years (Figure 2D). Women had a mean age of 71.7 ± 6.4 years (range: 60–94) and men of 73.1 ± 6.3 years (range: 60–93), a statistically significant difference (*p* = 0.0006). The proportion of patients with cognitive impairment at first visit was estimated using ACE-R and MMSE, both using adjusted thresholds as previously described (Butman et al., 2001; Sousa and Vivas, 2017). The proportion of the population meeting cognitive impairment criteria was 36.3% using MMSE and 40.9% using ACE-R, without differences between sexes, but a significant effect of age (OR = 1.1, *p* = 4×10^−11^) and years of education (OR = 0.91, *p* = 1.6×10^−4^). As expected, age was inversely correlated with schooling (Spearman’s *ρ* = −0.25, *p* < 0.001).

The executive function was assessed by IFS in the 26.1% of the present population, of which 86.6% showed scores below the threshold. After 2019, FAB was implemented, showing that 49.2% of the evaluated people had some executive dysfunction. These results support that FAB better aligns with the characteristics of the population. It is of note that despite the cognitive impairment, most patients preserved functionality, and only 19.0% patients met criteria for dementia, defined by the presence of cognitive impairment associated with functional dependency (CDR ≥ 1).

Neuroimaging abnormalities were frequent across the sample. Among cognitively impaired patients, over 90% presented abnormal MRI findings, with mixed degenerative and vascular pathology being the most common pattern. A notable proportion of cognitively normal patients also exhibited structural brain changes, highlighting the importance of neuroimaging even in the absence of cognitive symptoms.

A total of 20.0% of patients returned for at least one follow-up visit. Among those who had been cognitively normal at their initial evaluation, 25% showed evidence of cognitive decline at follow-up, underscoring the value of longitudinal monitoring in this population and the relevance of early detection strategies within the clinical framework of the Memory Clinic.

### 3.4 Cognitive reserve

**Figure 3:**
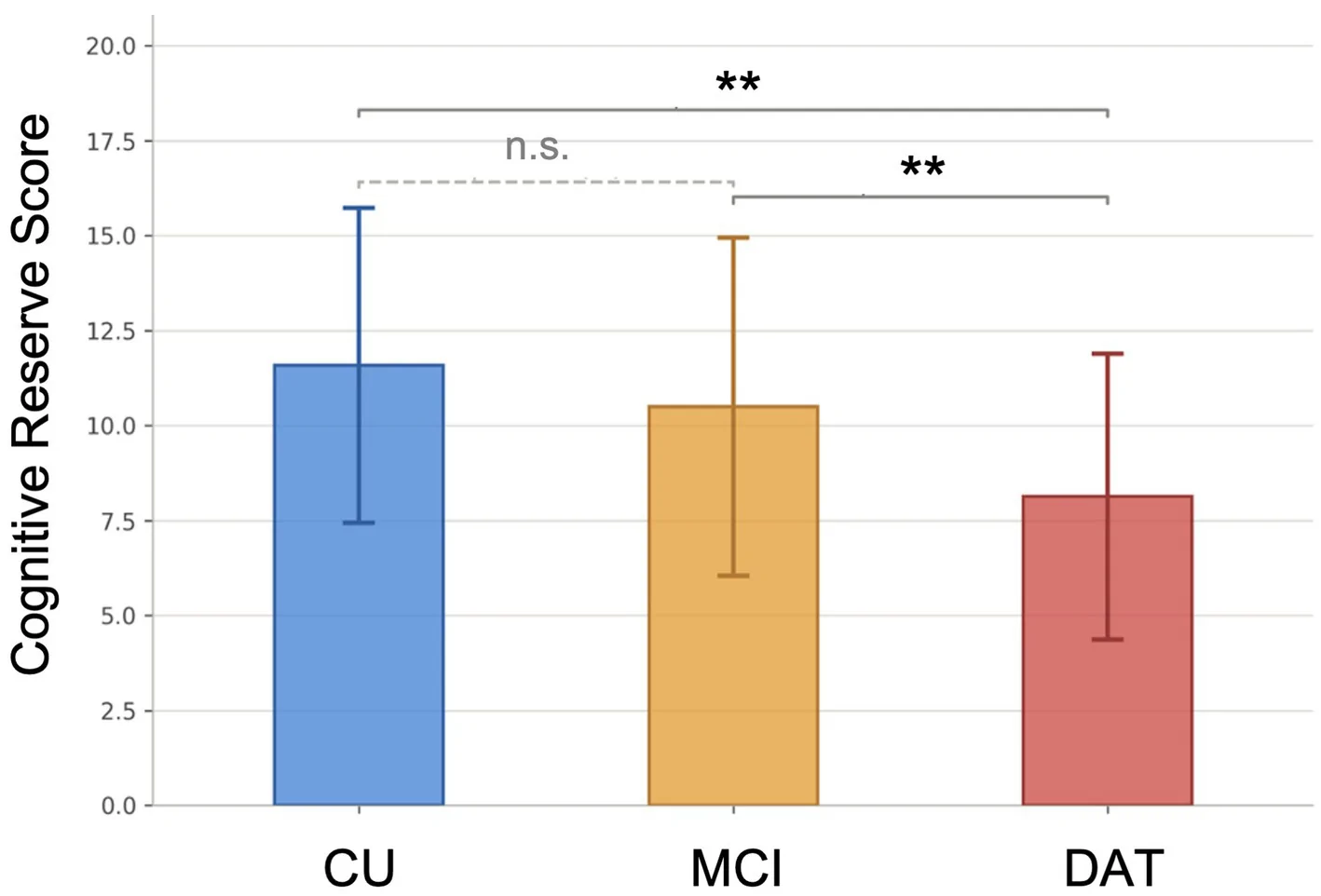
Cognitive reserve. Bar plot showing cognitive reserve scores in people cognitively unimpaired (CU), with mild cognitive impairment (MCI) and Alzheimer’s disease and related dementias (ADRD). Bars on top are showing groups being compared and its associated *p*-value estimated by PERMANOVA adjusted by sex, age, and years of education, followed by *post hoc* test Dwass–Steel–Critchlow–Fligner. **; *p* < 0.01; n.s., not significant (*p* > 0.05).

Cognitive reserve refers to the brain’s capacity to adapt and optimize cognitive functioning in response to aging, pathology, or injury, and is thought to reflect the flexibility and efficiency of neural networks involved in cognitive task execution. This reserve is typically measured through indirect proxy variables that capture life experiences accumulated over time (Stern, 2002). A questionnaire adapted to the present population was developed and implemented at the Memory Clinic, the CRQ-BA (see Methods). Using this instrument, cognitive reserve was evaluated in a subsample of 315 patients who attended the Memory Clinic between 2019 and 2023. Of these, 182 were cognitively normal, 79 had MCI, and 54 dementia. The sociodemographic characteristics of this subsample were consistent with those of the broader clinic population (73.0% women, mean age 72.4 ± 6.4 years; mean education 6.1 ± 3.1 years). The statistical model used showed that clinical diagnosis explained 5.7% of the variability in cognitive reserve scores (R^2^ = 0.057, *F*(2, 309) = 47.45, *p* = 0.001), with significant differences between people cognitively normal and those with dementia (*p* = 0.003), as well as between patients with MCI and dementia (*p* = 0.006). No significant differences were observed between cognitively normal participants and those with MCI (*p* = 0.147; Figure 3). These findings are consistent with previous studies (Liu et al., 2024).

Interestingly, beyond this association with dementia, CRQ-BA scores provide clinically meaningful information that complements formal cognitive testing. By capturing dimensions such as occupational history, social engagement, and lifestyle habits, it offers a broader picture of each patient’s accumulated life experiences and adaptive resources. This richer characterization of the individual supports more nuanced clinical judgment, allowing clinicians to better interpret cognitive test scores in context and to tailor follow-up and intervention strategies accordingly.

### 3.5 Cognitive stimulation therapy

CST has been endorsed by the National Institute for Health and Clinical Excellence (NICE) and Alzheimer’s Disease International (ADI), and has demonstrated positive effects on quality of life and communication skills, with favorable cost-effectiveness relative to pharmacological interventions (Lobbia et al., 2018; McDermott et al., 2019). This therapy consists of participation in a range of group activities and discussions aimed at improving cognitive and social functioning, and was found to be a feasible intervention in our population. The results of the pilot study were satisfactory for both patients and their families (Medel et al., 2024). To date, CST has been implemented four times in groups of approximately ten people with mild cognitive impairment (MCI) or mild dementia, with encouraging improvements in cognition observed in most participants (results in publication).

This intervention is currently conducted by the neuropsychologists of the Memory Clinic and recommended by neurologists to patients who meet the inclusion criteria. CST has become the complementary non-pharmacological therapy of choice in routine clinical practice at the Memory Clinic. We conducted an efficacy trial that yielded positive results on global cognitive functions (Medel et al., 2026).

### 3.6 Commitment with research

Practice-based research has been proposed as a critical bridge between clinical care and scientific knowledge, addressing research questions that arise directly from real-world practice and testing whether findings are feasible and effective in actual clinical settings (Cummings and Fulkerson, 2018). From its foundation, the Memory Clinic has embraced this principle, integrating clinical research as a core component of its activity alongside patient care. Since its inception, the clinic has contributed to a growing body of scientific literature spanning clinical medicine (Kochen et al., 2018; Parra et al., 2021), genetics (Dalmasso et al., 2019, 2024; Nicolas et al., 2025), biomarkers (Campanelli et al., 2025; Ramirez et al., 2025), and neuropsychology (Tassone et al., 2020; Medel et al., 2024), reflecting a sustained commitment to generating evidence from and for its own patient population. This ongoing research activity has not only advanced scientific knowledge but has directly informed and improved the clinical care provided at the clinic over time.

Since 2022, this research commitment has expanded through an active collaboration with Dr. M. C. Dalmasso and Dr. P. N. González, both researchers at the Studies in Neuroscience and Complex Systems Unit (ENyS-CONICET-HEC), to establish the Genetic and Neuroimaging Study of Aging and Dementia in the local population (GeNED.ar; https://enys.conicet.gov.ar/genedar/). This initiative aims to characterize the genetic, neuroimaging, and plasma profiles of aging and dementia in the local population, a community that remains critically underrepresented in large-scale dementia research cohorts. Crucially, this collaboration is conceived not as a unidirectional transfer of data from clinic to science, but as a bidirectional partnership: findings generated through GeNED.ar are actively integrated back into clinical practice at the Memory Clinic. A clear example is provided by a recent study analyzing plasma phosphorylated Tau at position 217 (pTau217) in 134 GeNED.ar participants (Ramirez et al., 2025). Two diagnostic models for the clinical application of pTau217 as a biomarker were proposed and evaluated, with results that were consistent with and supportive of the clinical diagnoses previously established by the Memory Clinic team. This convergence between biomarker-based research findings and clinical judgment exemplifies the translational potential of the GeNED.ar cohort, and underscores the Memory Clinic’s role not merely as a recruitment site, but as an active and equal partner in the generation and application of neuroscientific knowledge for the benefit of its patient population.

## 4 Discussion

This study describes ten years of operation of a Memory Clinic embedded within the public healthcare system of a socioeconomically vulnerable region of Argentina, providing a comprehensive account of its clinical, methodological, and research trajectory. The findings offer relevant insights into the challenges and opportunities of delivering specialized cognitive care to underserved populations.

A central observation is that the majority of patients attending the Memory Clinic did not meet criteria for dementia at the time of first consultation. Despite measurable cognitive deficits, present in approximately 40% of patients, only 19% fulfilled dementia criteria. This dissociation between cognitive and functional performance is particularly relevant in populations with low educational attainment and high levels of family and community integration, where compensatory mechanisms may preserve autonomy even in the presence of significant cognitive decline (Kochen et al., 2018). These findings are consistent with the concept of cognitive reserve, which has been shown to modulate the clinical expression of neurodegeneration (Liu et al., 2024). In our population, cognitive reserve was significantly lower in patients with dementia, further supporting its protective role and reinforcing the importance of contextualizing cognitive test results within each patient’s biographical and sociocultural background. The development and implementation of a locally adapted cognitive reserve instrument further illustrates the clinic’s commitment to context-sensitive, population-specific assessment.

The low overall educational level of the sample has direct implications for neuropsychological assessment. Standard cognitive instruments are sensitive to educational level, and their uncritical application in this population risks both overdiagnosis and underdiagnosis. The use of education-adjusted cut-off scores for the MMSE and ACE-R, is essential to ensure diagnostic accuracy. The transition from IFS to FAB for executive function assessment, illustrates the iterative refinement of the protocol in response to empirical observations. The substantially lower rate of impairment detected by FAB (49.2%) compared to IFS (86.6%) suggests that the latter overestimated executive dysfunction in this context, and that instrument selection must be guided by population characteristics rather than applied uniformly.

The significant difference in age at first presentation between women and men, combined with the absence of a significant sex difference in the proportion presenting with cognitive impairment, suggests that women attend earlier in the course of their cognitive trajectory rather than with lesser cognitive burden. This pattern may reflect greater health-seeking behavior in women, higher awareness of cognitive symptoms, or differential social dynamics in recognizing and acting upon memory complaints. These observations are consistent with broader literature on sex differences in dementia presentation and healthcare utilization (Ferretti et al., 2018), and have practical implications for clinical planning and early intervention strategies.

Neuroimaging findings were striking: over 90% of cognitively impaired patients presented abnormal MRI results, with mixed degenerative and vascular pathology. Based on previous observations reported in Argentina (Ferrante et al., 2016; Calandri et al., 2024), this high prevalence of structural brain changes likely reflects the cumulative impact of vascular risk factors associated with socioeconomic disadvantage in this population. Importantly, a notable proportion of cognitively normal patients also exhibited structural abnormalities, underscoring the value of neuroimaging as part of a comprehensive diagnostic workup even in the absence of overt symptoms. The availability of a 3 Tesla Philips Achieva scanner at HEC, and the systematic encouragement of patients to use this facility, has contributed to the quality and consistency of neuroimaging data, while also enabling the integration of standardized imaging protocols into the GeNED.ar research cohort.

The longitudinal data, though limited to 20% of patients who returned for follow-up, are clinically significant: 25% of those initially classified as cognitively normal showed evidence of decline at a subsequent visit. This conversion rate reinforces the importance of structured follow-up in populations at high social and vascular risk, and highlights the limitations of single-visit assessments. Expanding longitudinal monitoring remains a priority for the clinic’s future development.

The group cognitive stimulation program implemented in our setting has proven to be an effective non-pharmacological intervention for slowing cognitive decline, improving quality of life, and preserving functional abilities in patients with mild cognitive impairment and dementia. Nevertheless, despite the strong evidence supporting its benefits, the implementation of this program in our context is constrained by significant accessibility barriers. These include a shortage of specialized human resources, insufficient appropriate physical infrastructure within the public healthcare system, transportation difficulties faced by patients in reaching the center, and extreme weather conditions, including high temperatures and heavy rainfall, which collectively limit the universal reach of this resource. In response to this scenario, one potential alternative is the delivery of cognitive stimulation through virtual modalities. Although virtual Cognitive Stimulation Therapy (CST) has demonstrated effectiveness in other settings, its widespread adoption in our population is hindered by substantial obstacles, including limited internet access, low levels of digital literacy, and the scarcity of technological devices among the older adult and retired population that constitutes our patient base. Importantly, however, while these limitations are prevalent, they do not apply uniformly to all patients served. A promising path for future research would therefore involve the development of a pilot study aimed at implementing virtual CST in a subgroup of patients with internet access, appropriate devices, and basic digital competencies. Such an approach would enable the evaluation of the feasibility and acceptability of this intervention in our context, generating preliminary evidence to determine whether this modality could eventually be scaled to other patients who might derive benefit from it.

Memory clinics have been recognized as effective models for integrated dementia care (Lee et al., 2022). The model described here adopt these principles, tailoring them to the sociodemographic conditions and educational gradient of the population. This characterization enables higher-resolution clinical judgment, facilitating the interpretation of neurocognitive profiles within their appropriate context. Such precision enhances intervention strategies and is particularly critical in vulnerable settings.

The integration of clinical care with active research participation has contributed to staff training, protocol optimization, and alignment with international standards, while generating locally relevant evidence. Additionally, its integration into the public healthcare network has reduced the diagnostic gap previously affecting both physicians and the PAMI system (Kochen et al., 2018). As the only public Memory Clinic of its kind in the region, this center provides equitable access to high-quality cognitive care to a population that would otherwise lack it entirely, representing a potentially replicable model for similar settings across Latin America.

### 4.1 Limitations and methodological challenges

In addition to the specific barriers described for the cognitive stimulation program, other limitations affect the clinic’s capacity to reach the population it serves. As the only public center providing specialized cognitive care in the region, patients and families depend entirely on this single point of access, which cannot absorb the demand from surrounding areas. This centralization also limits the coverage of caregiver-directed interventions, a critical component that has so far been addressed only informally, through community outreach talks delivered during patient and family visits, but which has not yet been formally protocolized. Additional economic limitations affecting this population hinder regular attendance, follow-up, and adherence to recommended interventions. Limited internet access, lack of wifi or mobile devices, and low digital literacy among this predominantly older, low-educated, and retired population further restrict the feasibility of expanding remote or hybrid care models. Consistent with these limitations, only 20.0% of patients returned for at least one follow-up visit. This low rate reflects, at least in part, the same structural barriers described above, some of which could be addressed through the progressive incorporation of remote care strategies.

### 4.2 Future directions

International evidence supports the adoption of remote care modalities as a strategy to overcome these physical access barriers. Virtual delivery of Cognitive Stimulation Therapy has been shown to be a feasible and acceptable alternative to in-person delivery (Perkins et al., 2022), including in resource-limited settings comparable to ours (Fisher et al., 2024). However, our Memory Clinic currently lacks the technological infrastructure and the locally validated methodological adaptations required to implement this modality. Future work will therefore focus on the translation, cultural adaptation, and pilot testing of a virtual CST model for our population, building on the same adaptation methodology previously used for the in-person program.

Caregiver support represents a second priority area. Although caregiver burden is systematically assessed through the Zarit Burden Interview, our clinic does not yet offer a structured caregiver intervention. Virtual programs such as the Dementia Awareness Course have been successfully implemented in Brazil (de Carvalho et al., 2025; Fisher et al., 2024), and STrAtegies for RelaTives (START), a psychological intervention for family caregivers with demonstrated clinical effectiveness (Livingston et al., 2020a, 2020b), represent concrete models for adaptation to our setting. The translation, cultural adaptation, and pilot testing of these interventions, together with securing the institutional resources required for their implementation, constitutes a central line of future work.

Taken together, our immediate plan seeks to sustain the programs currently in place and advance their qualitative evolution toward a comprehensive, equitable, and sustainable model of care, so that scarcity of resources does not condition the right to dignified, humane, and high-quality neurocognitive care. Our research group remains committed to progressively overcoming these barriers in order to improve the wellbeing of the vulnerable communities served by the clinic.

## 5 Conclusion

This study documents ten years of operation of a Memory Clinic serving a socioeconomically vulnerable population in Florencio Varela, Argentina. Through the progressive refinement of standardized diagnostic protocols, the integration of interdisciplinary care, and a sustained commitment to practice-based research, the clinic has evolved from a gap-filling intervention into a stable and replicable model of specialized cognitive care within the public healthcare system. The results highlight the importance of adapting clinical tools and diagnostic criteria to the specific characteristics of low-education, high-vulnerability populations, and demonstrate that high-quality, research-integrated dementia care is both feasible and impactful in resource-limited settings. As dementia prevalence continues to rise disproportionately in low- and middle-income countries, the experience of this Memory Clinic offers a valuable and transferable framework for similar contexts across Latin America and beyond.

## Data Availability

The raw data supporting the conclusions of this article will be made available by the authors, without undue reservation.

## Footnotes

1
https://www.argentina.gob.ar/sites/default/files/informe_partido_de_florencio_varela.pdf
